# Valorization of Dairy and Fruit/Berry Industry By-Products to Sustainable Marinades for Broilers’ Wooden Breast Meat Quality Improvement

**DOI:** 10.3390/foods13091367

**Published:** 2024-04-28

**Authors:** Jolita Klementaviciute, Paulina Zavistanaviciute, Dovile Klupsaite, João Miguel Rocha, Romas Gruzauskas, Pranas Viskelis, Noureddine El Aouad, Elena Bartkiene

**Affiliations:** 1 Institute of Animal Rearing Technologies, Faculty of Animal Sciences, Lithuanian University of Health Sciences, Mickeviciaus Str. 9, LT-44307 Kaunas, Lithuania; jolita.klementaviciute@lsmu.lt (J.K.); paulina.zavistanaviciute@lsmu.lt (P.Z.); dovile.klupsaite@lsmu.lt (D.K.); 2 Department of Food Safety and Quality, Faculty of Veterinary, Lithuanian University of Health Sciences, Mickeviciaus Str. 9, LT-44307 Kaunas, Lithuania; 3 Universidade Católica Portuguesa, CBQF—Centro de Biotecnologia e Química Fina—Laboratório Associado, Escola Superior de Biotecnologia, Rua Diogo Botelho 1327, 4169-005 Porto, Portugal; jmfrocha@fc.up.pt; 4 LEPABE—Laboratory for Process Engineering, Environment, Biotechnology and Energy, Faculty of Engineering, University of Porto, Rua Dr. Roberto Frias, 4200-465 Porto, Portugal; 5 ALiCE—Associate Laboratory in Chemical Engineering, Faculty of Engineering, University of Porto, Rua Dr. Roberto Frias, 4200-465 Porto, Portugal; 6 Artificial Intelligence Centre, Kaunas University of Technology, K. Donelaicio Str. 73, LT-44249 Kaunas, Lithuania; romas.gruzauskas@ktu.lt; 7 Lithuanian Research Centre for Agriculture and Forestry, Institute of Horticulture, Kauno Str. 30, LT-54333 Babtai, Lithuania; pranas.viskelis@lammc.lt; 8 Laboratory of Life and Health Sciences, Faculty of Medicine and Pharmacy, Route de rabat km 15 Gzenaya BP 365 Tanger, University Abdelmalek Essaâdi, Tetouan 92000, Morocco; n.elaouad@uae.ac.ma

**Keywords:** broilers’ wooden breast meat, marinades, lactic acid bacteria, milk permeate, apple by-products, blackcurrant by-products, meat quality

## Abstract

**Simple Summary:**

Producers of poultry meat are confronted with significant challenges, including wooden breast meat (WBM) quality improvement. The study aims to improve the quality of WBM via the use of newly developed marinades based on selected strains of lactic acid bacteria (LAB) in combination with the by-products of the dairy and fruit/berry industries. These marinades would then be used for WBM quality enhancement. Six groups of marinades were prepared: Mp + Lc; Mp + Lc + ApBp; Mp + Lc + BcBp; Mp + Lu; Mp + Lu + ApBp; and Mp + Lu + BcBp. Further marinades were applied for broilers’ WBM pre-treatment. Non-treated WBM samples were analyzed as control. The results showed that, after 48 h of marination, enterobacteria and molds/yeasts in WBM were absent. Marinated (24 and 48 h) WBM showed lower dry-matter (DM) and protein content, as well as lower water holding capacity, and exhibited higher drip loss (on average, by 8.76%) and cooking loss (on average, by 12.3%), in comparison with controls. After WBM treatment, biogenic amines decreased; besides, the absence of spermidine and phenylethylamine was observed in meat marinated for 48 h with a marinade prepared with Lu. Overall, this research highlights the potential advantages of the developed sustainable marinades in enhancing the safety and quality attributes of the WBM.

**Abstract:**

The study aims to improve the quality of wooden breast meat (WBM) via the use of newly developed marinades based on selected strains of lactic acid bacteria (LAB) in combination with the by-products of the dairy and fruit/berry industries. Six distinct marinades were produced based on milk permeate (MP) fermented with *Lacticaseibacillus casei* (Lc) and *Liquorilactobacillus uvarum* (Lu) with the addition of apple (ApBp) and blackcurrant (BcBp) processing by-products. The microbiological and acidity parameters of the fermented marinades were evaluated. The effects of marinades on the microbiological, technical, and physicochemical properties of meat were assessed following 24 and 48 h of WBM treatment. It was established that LAB viable counts in marinades were higher than 7.00 log_10_ colony-forming units (CFU)/mL and, after 48 h of marination, enterobacteria and molds/yeasts in WBM were absent. Marinated (24 and 48 h) WBM showed lower dry-matter and protein content, as well as water holding capacity, and exhibited higher drip loss (by 8.76%) and cooking loss (by 12.3%) in comparison with controls. After WBM treatment, biogenic amines decreased; besides, the absence of spermidine and phenylethylamine was observed in meat marinated for 48 h with a marinade prepared with Lu. Overall, this study highlights the potential advantages of the developed sustainable marinades in enhancing the safety and quality attributes of WBM.

## 1. Introduction

Nowadays, producers are confronted with significant challenges, including alterations in the functional and technological attributes of raw meat as a result of contemporary intensive livestock fattening methods, among other factors [1,2,3]. Additionally, intense genetic selection and the adoption of intensive animal production systems for poultry growth have led to the emergence of anomalies (including white striping, wooden breast (WB) meat, deep pectoral muscle myopathy and pale, soft, exudative meat) in broilers’ chicken breast musculature [4,5,6,7]. Among myopathies garnering substantial attention from researchers and food technologists, the WBM anomaly stands out [5]. This condition is typified by a discernible rigidity that may impact various regions of the *Pectoralis major* [8]. WBM myopathy predominantly manifests as a conspicuous firmness in broilers’ chicken breast muscles, accompanied by morphometric and histopathological changes, as well as physicochemical irregularities, which can result in undesirable sensory, nutritional, physical chemical and technological characteristics [3,5,9]. Breast tissue affected by myopathy exhibits elevated levels of insoluble and total collagen compared to unaffected tissue [9], suggesting a potential link to increased tissue rigidity, reduced tenderness, and compromised meat quality. Structural changes significantly impact meat texture, pH, and water-holding capacity, potentially affecting microbial growth and safety, as well as shelf life [10,11]. Due to its unattractive appearance and texture, consumers typically have low acceptance of wooden breast meat (WBM) in its raw form, leading to its frequent use in minced meat products [12,13], such as sausages [14], patties [15], meatballs [16] or animal feed [12]. The incorporation of WBM into other products is not financially viable, as it is associated with a number of significant factors, including reduced productivity, meat processing challenges, and reduced consumer acceptance due to unfavorable sensory qualities [3,13]. Therefore, urgent scientific attention is warranted to develop cost-effective methodologies aimed at enhancing the quality of WB-afflicted meat [3]. Along with this, the employment of new marination techniques [17] can ameliorate raw WB properties. The use of natural marinades is a widely employed technique for meat pre-treatment and preservation [18,19]. Tailored marination strategies show promise in effectively managing meat quality issues associated with WB broilers’ chicken meat condition. Additionally, they have demonstrated the capacity to enhance the intensity of aromatic and flavor attributes while, simultaneously, reducing the chewiness, hardness and cohesiveness of meat [20,21,22].

It was reported that the lactic acid bacteria (LAB) strains *Pediococcus pentosaceus* and *Pediococcus acidilactici* could be successfully applied in the production of potato juice-based marinades for pork meat pre-treatment [23]. However, the preparation of LAB biomass and the preservation of its viability are essential steps if we want to use it in practical applications. Our previous studies showed that the LAB strains could be multiplied in the dairy industry by-product milk permeate (Mp) [24]. Additionally to the high viability of LAB in fermented milk permeate, the latter bio-product possesses desirable antimicrobial properties [25]. It was also reported that apple and blackcurrant by-products are very prospective antimicrobial food ingredients [26], which can be used in combination with fermented milk permeate, with the aim of functional properties improvement [27,28]. However, despite LAB having the capacity to metabolize amino acids in food, resulting in the production of desirable flavor and antimicrobial compounds, among others, they can also lead to non-desirable compounds, i.e., biogenic amine (BA) formation [29,30]. Finally, not only the sensory and technological characteristics of the marinated meat must be analyzed, but safety parameters, including BA concentration, should be taken into consideration.

The purpose of this study was to improve the quality of WBM via the use of newly developed marinades based on selected LAB strains [*Lacticaseibacillus casei* (Lc) and Liquorilactobacillus uvarum (Lu)] in combination with the by-products of the dairy (MP) and fruit/berry (Ap/BC) industries.

## 2. Materials and Methods

### 2.1. Materials Used for Experiment

A commercial processing company supplied broiler samples of the Ross 308 strain, acquired when the chicks were six weeks old. All broilers were raised in a deep litter under identical climate-controlled conditions and provided with the same standard feed. The hardness of the *Pectoralis major* muscle was assessed 6 h post-mortem, following the methodology outlined by Tijare et al. [31]. The study focused on selecting extremely hard and rigid samples spanning from the cranial region to the caudal tip of the fillets. Only fillets exhibiting consistent hardness ratings on both the left and right sides were considered for further analysis. Subsequently, for analytical purposes, the samples were vacuum-sealed and stored at a temperature of +4 °C until marination.

*Lacticaseibacillus casei* LUH210 (Lc) and *Liquorilactobacillus uvarum* LUHS245 (Lu) strains were sourced from the microorganism’s collection of the Lithuanian University of Health Sciences (Kaunas, Lithuania). Isolation, identification and phenotype characterization by PCR of LAB strains used in this experiment were described in previous studies by Bartkiene et al., 2020 [32]. These LAB strains were stored at −80 °C using a Microbank system (Pro-Lab Diagnostics, UK) and, subsequently, individually cultured in MRS broth with Tween 80 (Biolife, Milan, Italy) at 30 °C for a duration of 48 h prior to their use for milk permeate fermentation. The MP was obtained from the agricultural cooperative Pienas LT (Biruliskes, Lithuania).

Freeze-dried by-products of apple (variety Auksis) and blackcurrant (variety Ben Alder) were acquired from the Institute of Horticulture, Lithuanian Research Centre for Agriculture and Forestry (Babtai, Kaunas district, Lithuania).

### 2.2. Preparation of Marinades and Their Analyses

**Figure 1:**
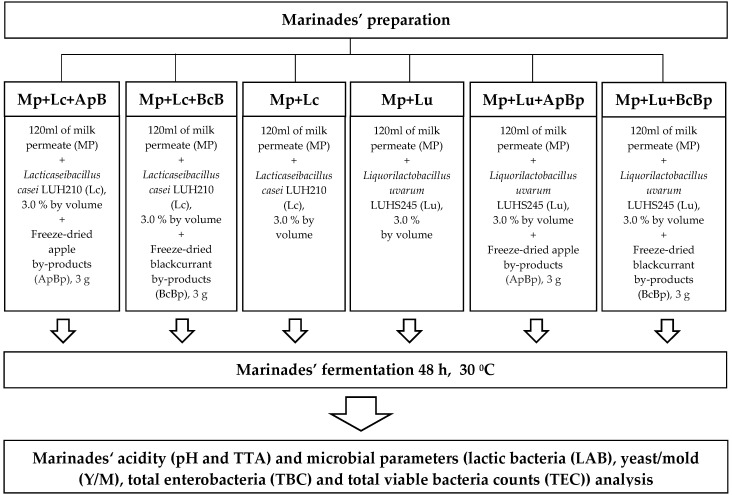
The principal scheme for marinade preparation (TTA—total titratable acidity; Mp—milk permeate; Lc—*Lc. casei*; Lu—*Liq. uvarum*; ApBp—apple by-products; BcBp—blackcurrant by-products).

A total of 3% (*v* (inoculum)/*v* (milk permeate)) of multiplied LAB (Lc and Lu, separately) with a cell concentration, on average, of 9.20 log_10_ colony-forming units (CFU)/mL were inoculated in Mp, followed by fermentation for 48 h at 30 ± 2 °C. Prior to fermentation, ApBp and BcBp by-products were added. Finally, six different marinades were prepared: Mp + Lc; Mp + Lc + ApBp; Mp + Lc + BcBp; Mp + Lu; Mp + Lu + ApBp; and Mp + Lu + BcBp. The following characteristics of the marinades were analyzed: pH, total titratable acidity (TTA), LAB, mold/yeast (M/Y), total enterobacteria (TEC), and total bacterial (TBC) viable counts. The principal scheme for the marinade preparation is given in Figure 1.

For LAB viable counts determination, the method described in ISO 15214:1998 for TBC assessment, ISO 4833-2:2013 for TEC analysis, ISO 21528-2:2017, and ISO 21527-2:2008 methods for M/Y evaluation were used [33,34,35,36]. The pH measurements of the marinades were acquired through the employment of a pH electrode (PP-15, Sartorius, Goettingen, Germany). The TTA was determined by homogenizing a 10 g sample (solution) with 90 mL of distilled water and quantifying it as the volume (mL) of 0.1 M NaOH (Sigma-Aldrich, Inc., St. Louis, MO, USA) solution required to achieve pH 8.2 (expressed in Neiman degrees, °N) [37].

### 2.3. Technology for Broilers’ Breast Meat Marination

In the second stage of the experiment, seven distinct groups of meat samples were prepared: the control group, denoted as WBM without any pre-treatment, and six experimental groups treated with different marinades (WBM + Mp + Lc; WBM + Mp + Lc + ApBp; WBM + Mp + Lc + BcBp; WBM + Mp + Lu; WBM + Mp + Lu + ApBp; and WBM + Mp + Lu + BcBp).

The immersion marination technique was used for sample pre-treatment: every set of samples was enclosed in a glass vessel, coated with a marinade and, subsequently, stored in a refrigerator at 4 ± 1 °C for 24 and 48 h.

**Figure 2:**
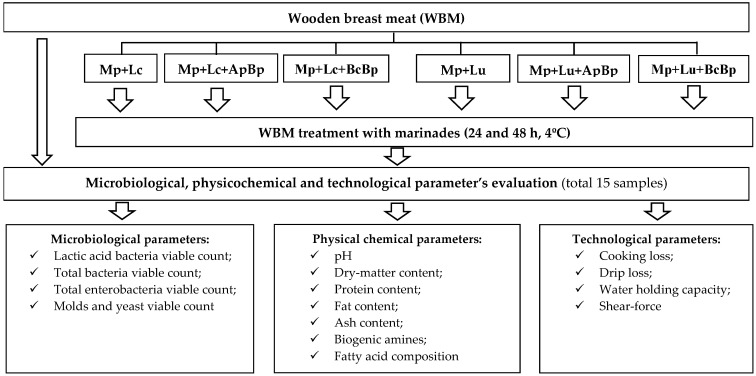
The principal scheme for broilers’ breast meat pre-treatment and meat quality parameter evaluation (WBM—wooden breast meat; Mp—milk permeate; Lc—*Lc. casei*; Lu*—Liq. uvarum*; ApBp—apple by-products; BcBp—blackcurrant by-products).

The following characteristics of WBM were analyzed: microbiological (LAB; TBC; TEC; and M/Y), physicochemical (pH; dry-matter (DM) content; protein content (PC); fat content (FC); ash content (AC); and fatty acid composition (FA)), and technological parameters (cooking loss (CL); drip loss (DL); WHC; and shear-force (SF)). The principal scheme for broilers’ breast meat marination and analysis is given in Figure 2.

### 2.4. Microbiological Parameters’ Evaluation Methods for Broilers’ Breast Meat

The microbiological parameters of the samples, including TBC, LAB, TEC, and M/Y counts, were evaluated. A 10 g and 10 mL sample was homogenized in 90 mL of a 0.9% sodium chloride solution for this evaluation. The sample was then prepared using saline serial dilutions ranging from 10^1^ to 10^7^. The M/Y viable counts were measured on Dichloran rose Bengal chloramphenicol agar (Liofilchem, Milan, Italy); TEC was measured on violet-red bile glucose agar (Oxoid Ltd., Basingstoke, UK); TBC was measured on plate count agar (Biolife, Milan, Italy); and LAB viable counts were measured on MRS agar with Tween-80 (Biolife, Milano, Italy). Section 2.2 provides standards for assessing microbiological parameters.

### 2.5. Main Physicochemical Parameters’ Evaluation of Broilers’ Breast Meat

Evaluation of the main physicochemical parameters of broiler meat samples encompassed the determination of meat pH, DM (%), FC (% of dry-matter), AC (% of dry-matter) and PC (% of dry-matter). Meat pH measurements were performed using an INOLAB3 pH-meter (WTW GmbH, Germany). DM was quantified in accordance with ISO 1442:2023 [38]. PC was determined through the evaluation of nitrogen content in adherence to ISO 937:2023 [39]. FC was determined in accordance with ISO 1443:2000 [40], which outlines the procedure for the assessment of total fat content in meat and meat products. The analysis of total ash content adhered to the ISO 936:1998 protocol specified for meat and meat products [41].

### 2.6. Methods for Meat Technological Parameters’ Evaluation

The WBM underwent analyses after 24 and 48 h of marination. The assessment of meat WHC, DL, CL and SF followed the methodologies described by Klupsaite et al. [42]. SF values were determined using a texture analyzer (TAXT2i version 6.06) equipped with a Warner-Bratzler shear blade and provided by Stable Micro Systems Co., Ltd., based in Goldaming, UK.

### 2.7. Method for Biogenic Amines’ Evaluation

The BAs, which encompass tryptamine (TRY), phenylethylamine (PHE), putrescine (PUTR), cadaverine (CAD), histamine (HIS), tyramine (TYR), spermidine (SPRMD) and spermine (SPRM), were analyzed in accordance with the methodology outlined in the publication by Ben-Gigirey et al. [43], with some modifications described by Bartkiene et al. [44]. Chromatographic analysis was carried out using a Varian ProStar HPLC system, manufactured by Varian Corp., based in Palo Alto, California, USA. The separation of amines was achieved through the utilization of a Discovery^®^ HS C18 column with dimensions of 150 mm × 4.6 mm-ϕ and a particle size of 5 µm-ϕ, provided by SupelcoTM Analytical located in Bellefonte, PA, USA. The identification of BA was conducted by comparing retention times with those of the established standards.

### 2.8. Analysis of Fatty Acid Profile

In accordance with the protocol described by Pérez-Palacios et al. [45], WBM lipids were extracted for the FA profile analysis using a combination of chloroform (Sigma-Aldrich, Inc., St. Louis, MO, USA) and methanol (2:1 *v*/*v*) (Sigma-Aldrich, Inc., St. Louis, USA). Then, using an esterification procedure of a 2 mol/L KOH solution (Sigma-Aldrich, Inc., St. Louis, USA) in methanol, fatty acid methyl esters (FAME) were produced. The FA composition was assessed using a gas chromatograph GC-2010 Plus (Shi-madzu Corporation, Tokyo, Japan), which was equipped with a mass spectrometer, GCMS-QP2010 (Shimadzu Corporation, Tokyo, Japan). Separation was executed on a Stabilwax-MS column 30 m, 0.25 mm ID, 0.25 µm provided by Restek Corporation, Bellefonte, PA, USA. The mass spectrometer operated in full scan mode. The oven temperature was programmed to start at 40 °C, climb by 8 °C/min to 220 °C, hold that temperature for 1 min, then increase by 20 °C/min to 240 °C, which was held for the final 10 min. The carrier gas used in the experiment was helium, which flowed at a rate of 0.91 mL/min. By comparing retention periods with the Supelco 37 Component FAME Mix reference material standard (Merck and Co., Inc., Kenilworth, NJ, USA), individual FAME peaks were identified.

### 2.9. Statistical Analysis

The data were analyzed using analysis of variance (ANOVA) and Tukey’s-honest significant difference (Tukey-HSD) as post-hoc tests using IBM SPSS^®^ Statistics 29 (IBM Corp., Armonk, New York, NY, USA) in order to assess the effects of various marinade compositions on WBM quality parameters, as well as the potential impacts of independently considered factors (LAB strain and fruit/berry industry by-products). In addition, a linear Pearson correlation was carried out using the statistical program SPSS to evaluate the degree of relationship between the variables. A *p*-value of 0.05 or less indicated statistical significance for the results (*p* ≤ 0.05).

## 3. Results and Discussion

### 3.1. Characteristics of the Developed Marinades

**Figure 3:**
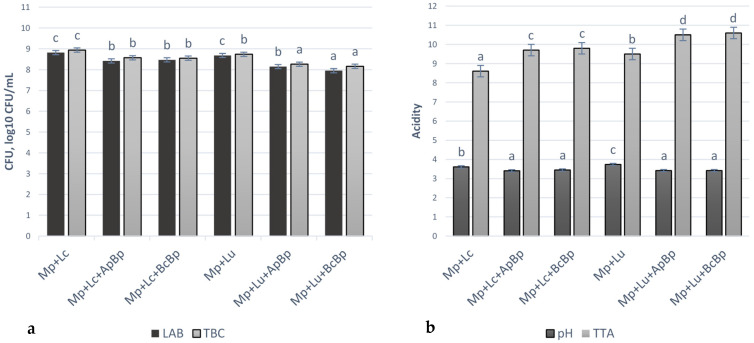
Microbiological (**a**) and acidity parameters (**b**) of marinades (LAB—lactic acid bacteria; TBC—total bacteria viable counts; CFU—colony-forming units; TTA– total titratable acidity, in Neiman degree (°N); Mp—milk permeate; Lc—*Lb. casei*; Lu—*Lb. uvarum*; ApBp—apple by-products; BcBp—blackcurrant by-products. ^a–d^ Mean values followed by the different superscript letter, are significantly different (*p* ≤ 0.05).

Microbiological and acidity parameters of marinades are shown in Figure 3. The TEC and M/Y were not observed in all tested marinades. The highest LAB viable counts were observed in the Mp + Lc and Mp + Lu groups (on average 8.75 ± 0.11 log_10_ CFU/mL). In other marinades, LAB viable counts were, on average, lower by 3.77% in Mp + Lc + ApBp, by 3.20% in Mp + Lc + BcBp, by 6.86% in Mp + Lu + ApBp and by 9.14% in Mp + Lu + BcBp, in comparison with Mp + Lc and Mp + Lu groups. The highest TBC was established in Mp + Lc marinades group (8.94 ± 0.06 log_10_ CFU/mL) and the lowest TBC was found in Mp + Lu + ApBp and Mp + Lu + BcBp groups (on average, 8.21 ± 0.05 log_10_ CFU/mL). A negative moderate correlation was found between LAB viable counts and TBC (r = −0.565, *p* < 0.001).

Marinades Mp + Lc + BcBp, Mp + Lc + ApBp, Mp + Lu + BcBp and Mp + Lu + ApBp showed the lowest pH values (on average, 3.43). In comparison with the latter samples, the pH of Mp + Lc and Mp + Lu groups was, on average, 5.12% and 8.42% lower, respectively. Negative moderate and negative very strong correlations were found between marinades pH and TTA (r = −0.593, *p* < 0.001), between pH and TBC (r = −0.728, *p* < 0.001), as well as between TBC and TTA (r = −0.930, *p* < 0.001). LAB strain, used for marinade preparation, was a significant factor for LAB viable counts (*p* = 0.035), TBC (*p* = 0.018) and for TTA (*p* = 0.006) of marinades. The type of fruit/berry industry by-product was a significant factor for LAB viable counts and TBC in marinades, besides pH and TTA (*p* ≤ 0.001).

Many studies have demonstrated the beneficial effects of *Lactobacillus* species against foodborne pathogens; however, not all LAB can be employed for meat fermentation since they differ in their mechanism of action and metabolite release [46,47,48,49,50,51,52,53,54]. A variety of compounds are produced by *Lactobacillus* spp., including lactic [55], formic, acetic, propionic, butyric, and succinic acids [56,57], ethanol, hydrogen peroxide, reuterin, antimicrobial peptides, bacteriocins, and bacteriocin-like inhibitory substances [58]. Additionally, the combination of LAB with fruit/berry by-products’ can lead to higher antimicrobial activity [24,25,26,28] because of the fruit/berry bioactive compounds, which inhibits pathogenic bacteria strains, encompassing both Gram-positive and Gram-negative types [59,60], as well as fungi [61]. Moreover, blackcurrant is acknowledged as a rich source of polyphenols, including anthocyanins, phenolic acid derivatives, flavanols and proanthocyanidins [62,63]. Our previous studies showed that blackcurrant inhibits *Salmonella enterica*, *Pseudomonas aeruginosa*, *Staphylococcus aureus*, *Enterococcus faecalis*, *Enterococcus faecium*, *Bacillus cereus*, *Streptococcus mutans*, *Staphylococcus epidermis*, *Staphylococcus haemolyticus* and *Pasteurella multocida* [28]. Apple pomace exhibits a significant presence of polyphenols, ranging from 31 to 51%, with a notable concentration of cinnamate esters, dihydrochalcones, and flavanols [64,65]. Our previous studies also showed that the antimicrobial properties of lyophilized blackcurrant and apple by-products can be enhanced in combination with the selected LAB strains [26].

### 3.2. Microbiological Parameters of Broilers’ Wooden Breast Meat

After 24 h of WBM marinating, the highest LAB viable counts were detected in WBM + Mp + Lu + BcBp group (7.29 ± 0.11 log_10_ CFU/mL) (Table 1). In other groups, the LAB viable counts were, on average, 14.8% (WBM + Mp + Lc), 3.99% (WBM + Mp + Lc + BcBp), 8.48% (WBM + Mp + Lu) and 10.6% (WBM + Mp + Lu + ApBp) lower, in comparison with the WBM + Mp + Lu + BcBp sample’s group. The type of fruit/berry industry by-product was a statistically significant factor for the LAB viable counts in WBM after 24 h of marinating (*p* < 0.001). After 48 h of treatment, LAB viable counts in all WBM samples were, on average, 7.31 ± 0.10 log_10_ CFU/mL. In comparison with 24 h treated WBM samples, 48 h marinated WBM showed, on average, 6.84% higher LAB viable counts.

The treated samples (24 and 48 h) showed, on average, 25.7 and 33.3 %, respectively, higher TBC viable counts, in comparison with the non-marinated. After 24 h of treatment, the highest TBC was found in the WBM + Mp + Lc + BcBp group (7.47 ± 0.01 log_10_ CFU/mL). LAB strain was a significant factor in TBC viable counts in WBM samples (*p* < 0.001). The 48-h marinated WBM + Mp + Lc + ApBp, WBM + Mp + Lc + BcBp, WBM + Mp + Lu + ApBp, and WBM + Mp + Lu + BcBp sample groups showed, on average, 7.72 ± 0.09 log_10_ CFU/mL TBC. A type of fruit/berry industry by-product was a significant factor on TBC viable counts in 48 h marinated WBM (*p* < 0.001).

In all cases, 24 h marination reduced TEC and M/Y viable counts. After 48 h of marinating, TEC and M/Y was not detected in any of the WBMs.

The variation in results observed across different treatments of samples can be attributed to several factors inherent to the microbiological characteristics of marinated products, particularly those derived from natural sources. Firstly, the proliferation *of Lactobacillus*, a crucial aspect of fermentation processes, is influenced by the capacity of LAB to extract energy from diverse advantageous compounds [55,66,67,68]. This metabolic activity is subject to environmental factors, such as food matrices and the presence of various interfering substances, which can significantly impact the survival and activity of specific LAB strains [69]. Studies on meat fermentation have reported a substantial increase in LAB viable counts, ranging from 3.00 to 4.00 log_10_ CFU/mL in raw meat to as high as 8.00 log_10_ CFU/mL [70,71,72]. Notably, both *Lb. casei* and *Lb. uvarum* have been found to effectively inhibit enterobacteria and mold/yeast during the fermentation process [69]. Furthermore, Gargi and Sengun discovered that incorporating probiotics, such as *Lacticaseibacillus rhamnosus*, *Lb. casei*, *Lactobacillus acidophilus* or their combination, after marination resulted in a significant reduction of *Salmonella typhimurium*, *Listeria monocytogenes* and *Escherichia coli* O157:H7 on the meat sample’s surface [73]. The initial viable counts of these bacteria (on average, 6 log_10_ CFU/mL) decreased to the range of 0.8–2.0, 2.1–3.3 and 0.7–2.7 log_10_ CFU/mL, respectively. Our previous studies showed that fruit/berry by-products, either individually [28] or in combination with selected LAB strains [24,26], have good antimicrobial properties. Fruits are a source of carbohydrates, organic acids, minerals, polyphenols, water-soluble vitamins (vitamin C and B-complex vitamins), provitamin A, amino acids, aromatic compounds, carotenoids, fibers, phytosterols and other bioactive substances [74], and berries contain a large amount of phenolic compounds, such as phenolic acids, flavonoids (flavanols), anthocyanins, tannins and ascorbic acid [75]. It was reported that apples, particularly organic peel and wild apple pomace oil, exhibit antimicrobial activity against numerous bacteria strains [76], including *B. cereus* and *E. coli* [77]. Apple’s compound phloretin shows antimicrobial properties inhibiting Gram-positive bacteria, in particular *S. aureus* ATCC 6538, *L. monocytogenes* ATCC 13932, methicillin-resistant *S. aureus* clinical strains, and *S. typhimurium* ATCC 13311 [78]. Miladinović et al. discovered that blackcurrant juices and extracts exhibited antimicrobial activity against a panel of foodborne and pathogenic microorganisms, and the most susceptible strains were *L. monocytogenes* and *P. aeruginosa* [79]. Kranz et al. reported that blackcurrant juice is very efficient at suppressing bacteria [80]. Additionally, when various antimicrobial agents are used in combination, it is crucial to choose the most appropriate combination so that favorable outcomes or even synergism can take effect, because different compounds have different antimicrobial mechanisms towards pathogen inhibition [26,47].

### 3.3. Chemical Composition and pH of Broilers’ Wooden Breast Meat

The chemical composition and pH of WBM are tabulated in Table 2. In comparison with non-treated, in all cases, marinated WBM samples showed significantly lower pH (on average, by 2.21% after 24 h of marination and by 6.19% after 48 h of marination). Comparing the 24 h marinated WBM groups, the lowest pH was obtained with WBM + Mp + Lc + ApBp group; in the other groups (WBM + Mp + Lc + BcBp, WBM + Mp + Lu + ApBp, and WBM + Mp + Lu + BcBp) the pH was, on average, 5.81 ± 0.05. After 48 h of treatment, the WBM + Mp + Lc and WBM + Mp + Lu + BcBp groups showed the lowest pH values (on average, 5.33 ± 0.02). After 24 h of marination, the lowest DM was attained in WBM + Mp + Lc + ApBp samples (21.3 ± 0.36%). On average, by 22.2 ± 0.20% higher DM was established in WBM + Mp + Lc, WBM + Mp + Lu and WBM + Mp + Lu +ApBp samples. The highest DM was observed in WBM + Mp + Lu + BcBp group (on average, by 1.2% higher, in comparison with WBM + Mp + Lc, WBM + Mp + Lu and WBM + Mp + Lu + ApBp).

The control sample’s DM was, on average, 3.3% higher, in comparison with 24 h marinated WBM and, on average, 2.2% higher, in comparison with 48 h marinated WBM. The pH and DM values of samples marinated for 24 h showed a significant positive correlation (r = 0.779, *p* < 0.001). The type of fruit/berry industry by-product was a significant factor for DM content in 24 and 48 h marinated WBM (*p* < 0.001 and *p* = 0.013, respectively). In comparison of the 24 and 48 h marinated samples with the control, on average, 2.93 and 1.97% lower PC was found in treated groups, respectively. After 24 h of treatment, WBM + Mp + Lc + ApBp samples showed the lowest PC content (17.4 ± 0.23%) and PC in WBM + Mp + Lc, WBM + Mp + Lc + BcBp, WBM + Mp + Lu, WBM + Mp + Lu + ApBp and WBM + Mp + Lu + BcBp groups was, on average, 2.0, 1.7, 2.1, 1.4, and 1.0 higher in comparison with WBM + Mp + Lc + ApBp, respectively. After 48 h of treatment, the WBM + Mp + Lu + ApBp group exhibited the highest PC (21.2 ± 0.14%) and PC in WBM + Mp + Lc, WBM + Mp + Lc + ApBp, WBM + Mp + Lc + BcBp, WBM + Mp + Lu and WBM + Mp + Lu + BcBp groups was, on average, 1.9, 1.5, 1.3, 2.1, and 2.0% lower in comparison with WBM + Mp + Lu + ApB, respectively. The type of fruit/berry industry by-products was a significant factor for PC in WBM (*p* < 0.001).

The stability of both meat and meat-derived products is intricately modulated by a multitude of variables, including, but not limited to, the specific composition and formulation of the marinade employed, along with the intricacies of treatment and the prevailing storage conditions [18,81,82]. Significant alterations in pH levels were discerned upon evaluating the impacts of the marination process. This is due to the fact that LAB can produce organic acids (among other compounds, for example CO_2_) which is, therefore, related to the environmental pH [83,84]. Xu et al., Yingying et al., Jing et al. and Fencioglu et al. detected a significant decrease in terms of pH value after the marination process [72,84,85,86]. DM changes occur due to the fact that the muscle tissue fluid possesses a lower ionic strength compared to the marinade solution, which enables the absorption of the marinade via osmotic processes until equilibrium is achieved [87]. Fencioglu et al. revealed that the marination process with different types of vinegar (balsamic, pomegranate, apple and grape) resulted in the absorption from 3.12 to 4.13% of the marinade liquids by the beef steak [85]. Furthermore, samples marinated with the probiotic *Lacticaseibacillus casei* exhibited high levels of satisfaction in terms of color, appearance, flavor and overall acceptability [73]. Wang et al. and Zhou et al. reported that proteins, the predominant compound of meat, undergo degradation and oxidation processes during the fermentation of meat [83,88]. The extent of reduction depended on the specific composition of the employed marinades. This was primarily due to marination and cooking-related factors, e.g., water evaporation, fat melting and protein loss [89]. Prolonged immersions of meat in marinating solutions can cause a significant protein loss in the liquid tissue, reaching up to 30% [90], thus diminishing the strength of the tissue structure. The degradation of numerous myofibrillar proteins through protein degradation, along with the reactive oxygen species-induced protein oxidation that damages myofibrillar proteins and activates the proteasome, collectively enhances the degradation of structural proteins in muscle, consequently improving meat tenderness [83,88,91].

### 3.4. Technological Characteristics of Broilers’ Wooden Breast Meat

Marination led to a higher CL of WBM (on average, 11.1 and 13.5% higher, after 24 and 48 h of marination, respectively). In comparison, CL of the 24 h marinated samples, WBM + Mp + Lu + BcBp exhibited the lowest values (on average, 24.9 ± 0.22%) (Table 3). Conversely, the highest CL was shown by the WBM + Mp + Lc + ApBp group (on average, 38.8%), thus representing, on average, 5.9 and 2.8% higher values in comparison with WBM + Mp + Lu + BcBp and WBM + Mp + Lc + BcBp groups, respectively. In comparison with the CL of the 48 h marinated samples, the WBM + Mp + Lc group exhibited the highest values (on average, 35.0 ± 0.45%), while other samples showed lower CL (on average, 5.3, 6.1, 2.4, 4.5 and 5.9% lower CL, respectively, in WBM + Mp + Lc + ApBp, WBM + Mp + Lc + BcBp, WBM + Mp + Lu, WBM + Mp + Lu + ApBp and WBM + Mp + Lu + BcBp groups). The LAB strain used for marinade preparation was a statistically significant factor for 24 h marinated WBM CL (*p* = 0.039); the type of fruit/berry industry by-product was a significant factor for 24 and 48 h marinated WBM CL (*p* = 0.002 and *p* < 0.001, respectively). A moderate negative correlation was established between 24 h marinated sample’s CL and DM values (r = −0.471, *p* < 0.01) and between CL and PC values (r = −0.557, *p* < 0.01).

Marination led to WBM WHC reduction and, on average, 4.88 and 7.12% lower WHC were found in 24 and 48 h marinated samples, respectively, in comparison with non-treated samples. The WBM + Mp + Lc + BcBp group exhibited the lowest WHC (58.1 ± 2.3%) after 24 h of marination. In contrast, WHC values of WBM + Mp + Lc, WBM + Mp + Lc + ApBp, WBM + Mp + Lu, WBM + Mp + Lu + ApBp and WBM + Mp + Lu + BcBp groups were higher (on average, by 4.60, 3.2, 6.30, 7.10, and 5.90%, respectively), in comparison with WBM + Mp + Lc + BcBp. After 48 h of marination, the highest WHC was attained in WBM + Mp + Lu samples (66.0 ± 0.9%), indicating, on average, 5.62% higher values in comparison with other treated groups. The LAB strain used for marinade preparation was a statistically significant factor for WBM WHC (*p* < 0.001). Positive moderate and strong correlations were found between WHC and PC values in 24 and 48 h marinated WBM (r = 0.568, *p* < 0.01 and r = 0.689, *p* < 0.001, respectively).

In comparison with non-marinated, 24 h treated samples showed, on average, 8.80%, and 48 h treated, on average, 8.72% higher DL. WBM + Mp + Lc and WBM + Mp + Lu + ApBp groups exhibited the lowest DL after 24 h of marination (on average, 6.66 ± 0.09%). After 48 h of marination, the lowest DL was found for WBM + Mp + Lc + BcBp, WBM + Mp + Lu and WBM + Mp + Lu + ApBp groups (averaging 6.52 ± 0.14%). In contrast, the WBM + Mp + Lc, WBM + Mp + Lc + ApBp and WBM + Mp + Lu + BcBp groups exhibited, on average, 2.30, 5.98 and 5.18%, respectively, higher DL in comparison with WBM + Mp + Lc + BcBp, WBM + Mp + Lu and WBM + Mp + Lu + ApBp groups. A strong negative correlation was found between WBM pH and DL (r = −0.692, *p* < 0.001). Besides, a positive correlation was established between 24 h marinated WBM DL and PC values (r = 0.630, *p* < 0.001).

The treatment has an impact on the variation in findings shown for each component examined. Significant changes in technological and sensory qualities, such as pH levels, cooking loss (CL), and shear force (SF), are caused by the myopathy that primarily affects meat quality [92,93,94,95]. There are two possible outcomes when meat’s pH is changed away from its isoelectric point: either an increase or decrease in water holding capacity (WHC). Raising the final pH is one way to counteract the detrimental effects of anomalies in broiler meat on the quality of the raw meat, but also makes it more difficult for the meat to absorb marinade solutions and hold moisture while cooking [4,96]. Research data from Xing et al. indicated a CL of about 17% in untreated WBM [12]. In contrast, studies undertaken by Mudalal et al., Madruga et al. and Zotte et al. reported higher percentages, ranging from 21–28% in terms of CL [4,14,97]. Gómez-Salazar et al. and Singh et al. observed that WHC is influenced by the composition of marinating solutions and the injection method used [98,99]. Samples subjected to fermentation and in which distinct marinade compositions, were employed displayed significantly reduced WHC. Many research studies have indicated that uncooked WBMs exhibit reduced WHC and elevated hardness [10,15,96]. In agreement with our studies, Mozuriene et al. discovered that pork meat marination (24 h) with lacto-fermented marinade lowered the WHC and, thus, increased cooking loss [23]. Latoch et al. reported that marinating pork steaks in fermented dairy products (kefir, yogurt and buttermilk) typically enhances the tenderness of meat, resulting in decreased hardness, particularly when cooked at temperatures of 60 or 80 °C for 6 h [100]. Zavistanaviciute et al. reported that incorporating *Lb. casei* and *Liq. uvarum* into marinades containing berry and fruit industry by-products resulted in enhanced WHC and increased overall acceptability of lamb meat [28].

### 3.5. Biogenic Amines’ Concentration in Marinated Broilers’ Wooden Breast Meat

The results of BA content in WBM are presented in Table 4. TRY, CAD and HIS were not detected in WBM. After 24 and 48 h of treatment, WBM samples demonstrated an absence of detectable PUTR, in contrast to the control group. In comparison with non-marinated WBM, 24 h treated WBM + Mp + Lc + ApBp, WBM + Mp + Lc + BcBp, WBM + Mp + Lu and WBM + Mp + Lu + BcBp groups showed, on average, 23.4% lower PHE concentration. After 48 h of marination, the WBM + Mp + Lu, WBM + Mp + Lu + ApBp, and WBM + Mp + Lu + BcBp groups disclosed, on average, 40.5% lower PHE content, in comparison with the control group. The lowest PHE concentration was found in the 24 h marinated WBM + Mp + Lu group (5.86 ± 0.27 mg/kg). The LAB strain used for marinade preparation was a statistically significant factor for PHE concentration in WBM (*p* < 0.001).

In comparison with the control group, WBM + Mp + Lc + ApBp, WBM + Mp + Lc + BcBp, WBM + Mp + Lu and WBM + Mp + Lu + ApBp samples exhibited, on average, 38.4% lower TYR concentration after 24 h of marination. Also, 48 h marinated WBM + Mp + Lc, WBM + Mp + Lc + ApBp, WBM + Mp + Lu + ApBp and WBM + Mp + Lu + BcBp samples exhibited, on average, 67.4% lower TYR content, in comparison with control group. Positive very strong correlation was found between 48 h marinated WBM pH and TYR concentration (r = 0.813, *p* < 0.001). The type of fruit/berry industry by-product was a statistically significant factor for TYR concentration in 24 h marinated WBM (*p* = 0.026). After 24 h of marination, the WBM + Mp + Lu group showed the lowest SPRMD content (20.0 ± 0.28 mg/kg). After 48 h of marination, SPRMD was not detected in the WBM + Mp + Lu, WBM + Mp + Lu + ApBp and WBM + Mp + Lu + BcBp groups. The LAB strain used for marinade preparation was a statistically significant factor in SPRMD concentration in marinated WBM samples (*p* < 0.001).

In comparison with non-treated samples, 24 h marinated WBM showed, on average, 19.65% lower SPRM content, and the lowest content was found in 24 h marinated WBM + Mp + Lu + ApBp samples (46.4 ± 0.77 mg/kg). Marination for 48 h reduced SPRM concentration in most of the WBMs (on average, by 53.50 ± 0.84 mg/kg, except WBM + Mp + Lc group). The lowest SPRM concentration was established in WBM + Mp + Lu + BcBp (46.2 ± 0.84 mg/kg). The LAB strain used for marinade preparation was a statistically significant factor for SPRM formation in 24 and 48 h marinated WBM (*p* < 0.001).

In fermented meat, the predominant BAs are TYR, CAD, PUTR and, to a lesser extent, HIS [30,101]. The accumulation of BAs in foods is contingent upon the availability of precursors, such as free amino acids [102,103,104], the presence of decarboxylase-positive non-starter microbiota, the composition of food, pH, ion strength and water activity of the raw-material, and conditions that favor the bacterial growth during food processing and storage [30,101,104,105,106,107,108]. As the pH decreases, there is an escalation in decarboxylase activity, leading to an increased production of BAs [101]. A number of techniques, including additives, bacterial starting cultures, oxidizing BAs, and temperature control, can be used to reduce the levels of BAs [109,110]. BA generation is influenced by fermentation and/or marination technique (marinade composition, process length, temperature, etc.) [111,112,113]. It has been documented that LAB treatment affects CAD and SPRM levels [114]. Through their competitive action against natural microbiota, starter cultures have been shown in numerous studies to have a role in lowering the accumulation of BAs in meat products [115,116,117]. The addition of *Staphylococcus xylosus* and *Lactiplantibacillus plantarum* effectively reduced TRY, PHE, PUTR, CAD, HIS and TYR by nearly 100, 100, 86, 63, 82, and 43%, respectively [116]. It was revealed, that *Lp. plantarum* is likely to reduce BA content through the action of BA oxidase and the inhibition of amine-producing microorganisms, which is facilitated by bacteriocin and other antibacterial metabolites [118]. Some strains of *Latilactobacillus sakei* subsp. *sakei* and *Lactiplantibacillus planatarum* have been shown to reduce the formation/accumulation of BAs [119].

### 3.6. Fatty Acid Profile of Broilers’ Wooden Breast Meat

The saturated fatty acid (SFA) profile (% of total fatty acid content) of marinated and control WBM is depicted in Table 5. The control group showed the highest SFA content (33.2%), in comparison to 24 and 48 h marinated groups (on average, by 2.91 and 2.91% lower, respectively). After 24 h of treatment, the SFA content was significantly the lowest in WBM + Mp + Lc + ApBp and WBM + Mp + Lc + BcBp groups, averaging 29.6 ± 0.32% compared to WBM + Mp + Lc and WBM + Mp + Lu groups.

Stearic acid (C18:0) and palmitic acid (C16:0) were the predominant SFAs in WBM. The control group showed the highest content of C16:0 (23.9%). However, after 24 and 48 h of treatment, C16:0 content was, on average, 2.93 and 2.52% lower, respectively. After 24 h of treatment, the WBM + Mp + Lc and WBM + Mp + Lc + ApBp groups had the lowest C16:0 content (on average, 20.1 ± 0.16%). In contrast, the WBM + Mp + Lc + BcBp, WBM + Mp + Lu, WBM + Mp + Lu + ApBp and WBM + Mp + Lu + BcBp groups showed, on average, 1.81, 2.10, 0.62 and 0.78% higher C16:0 content, respectively, in comparison with WBM + Mp + Lc and WBM + Mp + Lc + ApBp sample groups with 24 h of marination. After 48 h of marination, the lowest content of C16:0 was found in WBM + Mp + Lc and WBM + Mp + Lu + ApBp (20.57 ± 0.18%), and in WBM + Mp + Lc + ApBp, WBM + Mp + Lc + BcBp, WBM + Mp + Lu and WBM + Mp + Lu + BcBp groups, with values 0.32%, 1.84%, 1.84% and 0.79% higher, respectively, in comparison with WBM + Mp + Lc.

The LAB strain, used for marinade preparation, was a statistically significant factor for C12:0 (*p* < 0.001), C14:0 (*p* < 0.001), C15:0 (*p* = 0.008), C17:0 (*p* = 0.004) and C18:0 (*p* = 0.022) content in 24 h marinated WBM, and for C12:0 (*p* < 0.001), C14:0 (*p* < 0.001), C15:0 (*p* = 0.010) and C17:0 (*p* = 0.003) content in 48 h marinated WBM. Besides, the type of fruit/berry industry by-product was a significant factor for SFA content in WBM (*p* < 0.001).

Table 6 displays the monounsaturated fatty acid (MUFA) profile (% of total fatty acid content) of WBM samples. The predominant MUFAs were oleic acid (C18:1 ω9), palmitoleic acid (C16:1 ω7) and trans-vaccenic acid (C18:1 trans ω7). In 24 h marinated WBM samples, the C18:1 ω9 content was, on average, 0.97% higher in WBM + Mp + Lc, WBM + Mp + Lc + ApBp, WBM + Mp + Lc + BcBp, WBM +Mp + Lu and WBM + Mp + Lu + BcBp, in comparison with control samples. In 48 h marinated WBM samples, the C18:1 ω9 content was, on average, 1.34% higher in WBM + Mp + Lc + ApBp, WBM + Mp + Lc + BcBp, WBM + Mp + Lu, and WBM +Mp + Lu + ApBp groups, in comparison with the control WBM. The highest C18:1 ω9 content was found in WBM + Mp + Lc + BcBp group (in 24 and 48 h marinated samples, 36.6 ± 0.05 and 36.1 ± 0.03%, respectively). The lowest content of C18:1 trans ω7 was found in the WBM + Mp + Lu + BcBp group (1.55 ± 0.01%). After 24 h of marination, C18:1 trans ω7 content was, on average, 0.32% lower, in comparison with the control. Following 48 h of marination, C18:1 trans ω7 content was, on average, 0.37% higher in the control group, when compared to the WBM + Mp + Lc, WBM + Mp + Lc + ApBp, WBM + Mp + Lu + ApBp and WBM + Mp + Lu + BcBp groups. The LAB strain used for marinade preparation was a statistically significant factor for C14:1 (*p* = 0.043), C18:1 ω9 (*p* = 0.039) and C18:1trans ω7 (*p* = 0.005) content in WBM FA profile after 24 h of marination, and for C14:1 (*p* = 0.002) after 48 h of marination. Besides, the type of fruit/berry industry by-product was a significant factor for MUFA (*p* = 0.006) and C18:1 ω9 (*p* = 0.002) contents in 24 h marinated WBM.

Contrasting with non-marinated and treated samples, higher polyunsaturated fatty acid (PUFA) content was found in 24 and 48 h marinated samples (3.04 and 3.33%, respectively) (Table 7). After 24 h of marination, the highest PUFA content (on average, 30.74 ± 0.11%) was found in WBM + Mp + Lc + ApBp and WBM + Mp + Lu + ApBp groups. However, after 48 h of treatment, the highest PUFA content was found in WBM + Mp + Lu + ApBp group (on average, 30.03 ± 0.19%). Linoleic acid (C18:2 ω6) and α-linolenic acid (C18:3α ω3) were the predominant PUFAs in WBM. Furthermore, dihomo-gamma-linolenic acid C20:3 ω6, arachidonic acid C20:4 ω6 and eicosapentaenoic acid C20:5 ω3, three highly unsaturated fatty acids (HUFA), were found in WBM. The highest levels of HUFA were found in the WBM + Mp + Lc and WBM + Mp + Lu groups (on average, 0.86 ± 0.18%). 

In comparison with the control samples, 24 and 48 h marinated WBM showed, on average, 3.05 and 3.27% higher C18:2 ω6 content, respectively. After 24 h of marination, the highest C18:2 ω6 concentration was found in WBM + Mp + Lc + ApBp group (28.8 ± 0.05%), which was, on average, 1.84% higher than that in WBM + Mp + Lc, WBM + Mp + Lc + ApBp, WBM + Mp + Lu, WBM + Mp + Lu + ApBp and WBM + Mp + Lu + BcBp groups. In comparison, 48 h marinated samples, WBM + Mp + Lc + ApBp and WBM + Mp + Lu + ApBp showed the highest C18:2 ω6 content (on average, 28.7 ± 0.03%).

The type of fruit/berry industry by-product was a statistically significant factor for C18:2 ω6 (*p* = 0.003), C18:3α ω3 (*p* = 0.009), C20:3 ω6 (*p* = 0.012), C20:4 ω6 (*p* = 0.019) and PUFA (*p* = 0.006) content in 24 h marinated samples. Moreover, the type of fruit/berry industry by-product was a significant factor for C18:2 ω6 (*p* = 0.033), C18:3α ω3 (*p* < 0.001), C18:3γ ω6 (*p* < 0.001) and PUFA (*p* = 0.050) content in 48 h marinated samples. The LAB strain, used for marinades preparation, was a statistically significant factor for C18:3γ ω6 (*p* = 0.012), C20:3 ω6 (*p* = 0.035), C20:4 ω6 (*p* = 0.030) and C20:5 ω3 (*p* < 0.001) contents after 24 h, and C20:3 ω6 (*p* = 0.008), C20:4 ω6 (*p* < 0.001) and C20:5 ω3 (*p* = 0.042) contents in 48 h marinated WBM.

Depending on how the samples were treated, there are a number of reasons for the differences in the results for each factor that was evaluated. First off, when comparing WBM to poultry meat that is not affected, most studies show that WBM has higher levels of monounsaturated fatty MUFAs and lower levels of PUFAs and SFAs [120,121,122,123]. This divergence highlights WBM’s different lipid makeup and possible effects on meat quality. Breast myopathies are highly related to oxidative stress in the breast muscles of broiler chickens [121,124]. In impacted broiler breasts, lipid peroxidation products are accurate indicators of exposure to free radicals [121,125,126]. According to Jongberg et al., antioxidants are essential in preventing the oxidation of lipids and proteins because they provide hydrogen atoms from phenolic groups [127]. Therefore, using antioxidants that are found in plants naturally presents a viable way to reduce lipid oxidation and increase the shelf life of poultry meat [128,129]. Probiotics and fermented dairy products also have antioxidant qualities that help reduce the hazards associated with reactive oxygen species by breaking down hydrogen peroxide and peroxide anions [130]. By degrading hydrogen peroxide and peroxide anions, they reduce the risks related to reactive oxygen. Our previous works showed that the combination of *Lp. plantarum* (LUHS 135) strain and *Thymus vulgaris* essential oil used for lamb meat pre-treatment increased the concentration of PUFA in meat [41]. Changes in meat lipidomic profile may also be influenced by the lipolytic activity observed in LAB [131]. According to Tkacz et al., marinating affected the composition of FA in sous-vide beef, especially the oleic and palmitic FA [132]. Additionally, the overall SFA reduced, with the exception of the n-6/n-3 ratio. Furthermore, our previous research revealed that the FA content of lamb meat was affected by the addition of by-products from the fruit and berry industry to marinades [133]. The oil extracted from blackcurrant seeds is valued for having a desirable n-6/n-3 ratio and a high amount of PUFAs [111,134]. Apple oil includes substantial levels of C18:2 (55.5–57.8%) and C18:1 (25.5–29.4%) [76,134]. Additionally, α-linolenic C18:3 (54.3%) is abundant in oils extracted from by-products of *Malus* spp. (wild apple) [76].

## 4. Conclusions

The results of this study showed that recently developed marinades had great LAB viability; marinade formulations with compositions Mp + Lc and Mp + Lu showed the highest LAB viability, with an average of 8.75 log_10_ CFU/mL. Marinades proved to be effective in improving WBM’s microbiological safety. In particular, it was found that marinade compositions reduced WBM’s TEC and M/Y viable counts after 24 h of treatment and that they were completely eliminated after 48 h. Marinated WBM samples, in comparison to control, showed significantly lower pH (by 2.21 and 6.19%), DM (by 3.3 and 2.2%), PC (by 2.93 and 1.97%), and WBM (by 4.88 and 7.12%), and with significantly higher CL (by 11.1 and 13.5%), and DL (by 8.80 and 8.72%) after 24 and 48 h of marination, respectively. After WBM treatment, BA decreased; in addition, the absence of spermidine and phenylethylamine was observed in meat marinated for 48 h with a marinade prepared with Lu. Natural marinades containing selected LAB strains fermented with dairy and fruit/berry industry by-products could help solve WMB problems related to biogenic amines and microbiological safety. In marinades, industrial by-products can help reduce the cost of processing and their use for sustainability. In addition, these newly developed marinades can benefit the poultry industry by improving product quality, safety, and marketability. Future research may investigate the synergistic benefits of combining ApBp and BcBp in marinade formulations and incorporate sensory analysis to assess marinades’ compatibility for poultry meat.

## Figures and Tables

**Table 1:** Microbiological parameters (mean values ± standard errors) of broilers’ wooden breast meat.

Microorganism (log_10_ CFU/mL)				
Samples	LAB	TBC	TEC	M/Y
WBM	3.98 ± 0.05 ^φ^	5.06 ± 0.21 ^φ^	3.87 ± 0.06 ^φ^	3.69 ± 0.16 ^φ^
After 24 h of marinating				
WBM + Mp + Lc	6.35 ± 0.09 ^a,A,β^	7.15 ± 0.07 ^c,A,β^	3.54 ± 0.05 ^b,β^	3.17 ± 0.10 ^ab,β^
WBM + Mp + Lc + ApBp	6.95 ± 0.10 ^cd,A,β^	7.18 ± 0.01 ^c,A,β^	3.47 ± 0.05 ^b,β^	3.02 ± 0.09 ^a,β^
WBM + Mp + Lc + BcBp	7.01 ± 0.11 ^d,A,β^	7.47 ± 0.01 ^d,A,β^	3.16 ± 0.07 ^a,β^	3.29 ± 0.10 ^b,β^
WBM + Mp + Lu	6.72 ± 0.08 ^bc,A,β^	6.70 ± 0.05 ^b,A,β^	3.22 ± 0.03 ^a,β^	3.00 ± 0.07 ^a,β^
WBM + Mp + Lu + ApBp	6.59 ± 0.07 ^ab,A,β^	6.18 ± 0.06 ^a,A,β^	3.15 ± 0.09 ^a,β^	3.25 ± 0.04 ^b,β^
WBM + Mp + Lu + BcBp	7.29 ± 0.11 ^e,A,β^	6.90 ± 0.07 ^b,A,β^	4.41 ± 0.10 ^c,φ^	3.11 ± 0.09 ^ab,β^
After 48 h of marinating				
WBM + Mp + Lc	7.41 ± 0.06 ^a,B,β^	7.41 ± 0.01 ^a,B,β^	nd	nd
WBM + Mp + Lc + ApBp	7.30 ± 0.11 ^a,B,β^	7.67 ± 0.04 ^b,B,β^	nd	nd
WBM + Mp + Lc + BcBp	7.32 ± 0.10 ^a,B,β^	7.83 ± 0.07 ^b,B,β^	nd	nd
WBM + Mp + Lu	7.19 ± 0.13 ^a,B,β^	7.28 ± 0.10 ^a,B,β^	nd	nd
WBM + Mp + Lu + ApBp	7.25 ± 0.06 ^a,B,β^	7.64 ± 0.07 ^b,B,β^	nd	nd
WBM + Mp + Lu + BcBp	7.39 ± 0.05 ^a,B,β^	7.73 ± 0.06 ^b,B,β^	nd	nd

LAB—lactic acid bacteria; TBC—total bacteria viable counts; TEC—total enterobacteria viable counts; M/Y—mold and yeast viable counts; CFU—colony-forming units; WBM—wooden breast meat; MP—milk permeate; Lc—*Lc. casei*; Lu—*Liq. uvarum*; ApBp—apple by-products; BcBp—blackcurrant by-products; nd—not detected; ^a–e^ Mean values followed by the different superscript letter in the column are significantly different (*p* ≤ 0.05) between treatment groups in the same time duration; ^A,B^ Mean values followed by the different superscript letter in the column are significantly different (*p* ≤ 0.05) between treatment groups in different marination durations; ^φ,β^ Mean values followed by the different superscript letter in the column are significantly different from the control group (*p* ≤ 0.05); data expressed as the mean value (*n* = 3) ± standard error (SE).

**Table 2:** Chemical Composition and pH (mean values ± standard errors) of Broilers’ Wooden Breast Meat.

Samples	pH	DM, %	PC, %	FC, %	AC, %
WBM	5.89 ± 0.04 ^φ^	25.7 ± 0.28 ^φ^	21.7 ± 0.24 ^φ^	2.51 ± 0.06 ^φ^	1.47 ± 0.08 ^φ^
After 24 h of marinating					
WBM + Mp + Lc	5.75 ± 0.03 ^ab,B,β^	22.3 ± 0.23 ^b,A,β^	19.1 ± 0.25 ^cd,A,β^	2.01 ± 0.11 ^a,A,β^	1.19 ± 0.01 ^a,A,β^
WBM + Mp + Lc + ApBp	5.61 ± 0.06 ^a,A,β^	21.3 ± 0.36 ^a,A,β^	17.4 ± 0.23 ^a,A,β^	2.52 ± 0.14 ^b,A,φ^	1.36 ± 0.18 ^ab,A,φ^
WBM + Mp + Lc + BcBp	5.81 ± 0.04 ^b,B,φ^	23.4 ± 0.14 ^c,A,β^	19.5 ± 0.33 ^cd,A,β^	2.66 ± 0.10 ^b,A,φ^	1.22 ± 0.11 ^ab,A,β^
WBM + Mp + Lu	5.74 ± 0.07 ^ab,A,β^	22.1 ± 0.26 ^b,A,β^	18.8 ± 0.21 ^bc,A,β^	2.00 ± 0.18 ^a,A,β^	1.30 ± 0.05 ^ab,A,β^
WBM + Mp + Lu + ApBp	5.82 ± 0.05 ^b,B,φ^	22.1 ± 0.12 ^b,A,β^	18.4 ± 0.12 ^b,A,β^	2.30 ± 0.14 ^ab,A,φ^	1.38 ± 0.11 ^abA,φ^
WBM + Mp + Lu + BcBp	5.81 ± 0.06 ^b,B,φ^	23.4 ± 0.42 ^c,A,β^	19.4 ± 0.24 ^cd,A,β^	2.38 ± 0.11 ^b,A,φ^	1.57 ± 0.20 ^b,A,φ^
After 28 h of marinating					
WBM + Mp + Lc	5.35 ± 0.02 ^a,A,β^	23.1 ± 0.22 ^ab,B,β^	19.3 ± 0.18 ^ab,A,β^	2.50 ± 0.15 ^ab,B,φ^	1.29 ± 0.09 ^ab,A,φ^
WBM + Mp + Lc + ApBp	5.74 ± 0.09 ^c,A,β^	23.6 ± 0.31 ^bc,B,β^	19.7 ± 0.23 ^bc,B,β^	2.75 ± 0.12 ^b,A,φ^	1.11 ± 0.12 ^a,A,β^
WBM + Mp + Lc + BcBp	5.64 ± 0.03 ^bc,A,β^	24.2 ± 0.16 ^cd,A,β^	19.9 ± 0.26 ^bc,A,β^	3.16 ± 0.16 ^c,B,β^	1.14 ± 0.07 ^ab,A,β^
WBM + Mp + Lu	5.71 ± 0.05 ^c,A,β^	22.9 ± 0.24 ^a,B,β^	19.1 ± 0.22 ^a,A,β^	2.40 ± 0.19 ^ab,B,φ^	1.42 ± 0.15 ^b,A,φ^
WBM + Mp + Lu + ApBp	5.58 ± 0.03 ^b,A,β^	24.6 ± 0.19 ^d,B,β^	21.2 ± 0.14 ^d,B,β^	2.23 ± 0.13 ^a,A,φ^	1.24 ± 0.09 ^ab,A,β^
WBM + Mp + Lu + BcBp	5.32 ± 0.02 ^a,A,β^	22.9 ± 0.16 ^a,A,β^	19.2 ± 0.16 ^ab,A,β^	2.61 ± 0.17 ^ab,A,φ^	1.14 ± 0.13 ^ab,A,β^

WBM-wooden breast meat; Mp-milk permeate; Lc-*Lc. casei*; Lu*-Liq. uvarum*; ApBp-apple by-products; BcBp-blackcurrant by-products. DM-dry matter content; PC-protein content; FC-fat content; AC-ash content; ^a–d^ Mean values followed by the different superscript letter in the column are significantly different (*p* ≤ 0.05) between treatment groups for the same time duration; ^A,B^ Mean values followed by a different superscript letter in the column are significantly different (*p* ≤ 0.05) between treatment groups in different marination duration; ^φ,β^ Mean values followed by a different superscript letter in the column are significantly different from the control group (*p* ≤ 0.05); data expressed as the mean value (*n* = 3) ± standard error (SE).

**Table 3:** Technological characteristics (mean values ± standard errors) of broilers’ wooden breast meat.

Parameter				
Samples	CL, %	WHC, %	SF, kg cm^−2^	DL, %
WBM	17.43 ± 0.12 ^φ^	67.5 ± 0.20 ^φ^	1.85 ± 0.16 ^φ^	2.09 ± 0.09 ^φ^
After 24 h of marinating				
WBM + Mp + Lc	30.2 ± 0.57 ^cd,A,β^	62.7 ± 1.1 ^b,B,β^	1.64 ± 0.13 ^a,A,φ^	6.75 ± 0.12 ^a,A,β^
WBM + Mp + Lc + ApBp	30.8 ± 0.88 ^d,A,β^	61.3 ± 0.7 ^b,A,β^	1.67 ± 0.09 ^a,A,φ^	13.4 ± 0.14 ^d,B,β^
WBM + Mp + Lc + BcBp	28.0 ± 0.21 ^b,A,β^	58.1 ± 2.3 ^a,A,β^	1.68 ± 0.10 ^a,A,φ^	8.04 ± 0.16 ^b,B,β^
WBM + Mp + Lu	28.6 ± 0.96 ^bc,A,β^	64.4 ± 2.0 ^b,A,β^	1.71 ± 0.13 ^a,A,φ^	8.86 ± 0.09 ^c,B,β^
WBM + Mp + Lu + ApBp	28.7 ± 0.29 ^bc,A,β^	65.2 ± 1.7 ^b,B,β^	1.76 ± 0.11 ^a,Aφ^	6.57 ± 0.06 ^a,A,β^
WBM + Mp + Lu + BcBp	24.9 ± 0.22 ^a,A,β^	64.0 ± 1.6 ^b,B,β^	1.70 ± 0.08 ^a,A,φ^	9.15 ± 0.16 ^c,A,β^
After 48 h of marinating				
WBM + Mp + Lc	35.0 ± 0.45 ^d,B,β^	58.2 ± 1.4 ^a,A,β^	1.62 ± 0.07 ^a,A,φ^	8.55 ± 0.6 ^b,B,β^
WBM + Mp + Lc + ApBp	29.7 ± 0.57 ^ab,A,β^	60.8 ± 1.4 ^a,A,β^	1.66 ± 0.10 ^a,A,φ^	12.5 ± 0.10 ^d,A,β^
WBM + Mp + Lc + BcBp	28.9 ± 0.27 ^a,B,β^	58.7 ± 2.5 ^a,A,β^	1.65 ± 0.05 ^a,A,φ^	6.25 ± 0.14 ^aA,β^
WBM + Mp + Lu	32.6 ± 0.62 ^c,B,β^	66.0 ± 0.9 ^b,A,β^	1.67 ± 0.07 ^a,A,φ^	6.42 ± 0.16 ^a,A,β^
WBM + Mp + Lu + ApBp	30.5 ± 0.85 ^b,B,β^	59.0 ± 0.9 ^a,A,β^	1.65 ± 0.04 ^a,A,φ^	6.88 ± 0.13 ^a,B,β^
WBM + Mp + Lu + BcBp	29.1 ± 0.46 ^ab,B,β^	59.1 ± 1.5 ^a,A,β^	1.63 ± 0.06 ^a,A,φ^	11.7 ± 0.09 ^c,B,β^

WBM-wooden breast meat; Mp-milk permeate; Lc-*Lc. casei*; Lu*-Liq. uvarum*; ApBp-apple by-products; BcBp-blackcurrant by-products; CL-cooking loss; DL-drip loss; WHC-water holding capacity; SF-shear force; ^a–d^ Mean values followed by a different superscript letter in the column are significantly different (*p* ≤ 0.05) between treatment groups for the same time duration; ^A,B^ Mean values followed by a different superscript letter in the column are significantly different (*p* ≤ 0.05) between treatment groups for different marination durations; ^φ,β^ Mean values followed by a different superscript letter in the column are significantly different from the control group (*p* ≤ 0.05); data expressed as mean value (*n* = 3) ± standard error (SE).

**Table 4:** Biogenic amine content (mean values ± standard errors) (mg/kg) in broilers’ wooden breast meat.

Biogenic Amines, mg/kg								
	TRY	PHE	PUTR	CAD	HIS	TYR	SPRMD	SPRM
WBM	nd	8.19 ± 0.17 ^φ^	28.7 ± 0.61 ^φ^	nd	nd	16.8 ± 0.74 ^φ^	29.13 ± 0.77 ^φ^	65.63 ± 2.05 ^φ^
After 24 h of marinating								
WBM + Mp + Lc	nd	8.47 ± 0.12 ^c,B,β^	nd	nd	nd	16.8 ± 0.25 ^e,B,φ^	41.5 ± 0.47 ^e,B,β^	54.5 ± 0.55 ^c,A,β^
WBM + Mp + Lc + ApBp	nd	6.35 ± 0.28 ^ab,B,β^	nd	nd	nd	11.3 ± 0.30 ^c,B,β^	31.6 ± 0.56 ^c,B,β^	60.8 ± 0.87 ^d,B,β^
WBM + Mp + Lc + BcBp	nd	6.69 ± 0.13 ^b,B,β^	nd	nd	nd	12.5 ± 0.19 ^d,A,β^	35.1 ± 0.51 ^d,B,β^	55.2 ± 0.94 ^c,A,β^
WBM + Mp + Lu	nd	5.86 ± 0.27 ^a,A,β^	nd	nd	nd	9.17 ± 0.17 ^b,A,β^	20.0 ± 0.28 ^a,A,β^	49.0 ± 0.67 ^b,A,β^
WBM + Mp + Lu + ApBp	nd	8.03 ± 0.15 ^c,A,φ^	nd	nd	nd	8.44 ± 0.09 ^a,A,β^	30.5 ± 0.36 ^c,A,β^	46.4 ± 0.77 ^a,A,β^
WBM + Mp + Lu + BcBp	nd	6.19 ± 0.19 ^ab,A,β^	nd	nd	nd	18.6 ± 0.12 ^f,B,β^	27.2 ± 0.37 ^b,A,β^	50.5 ± 0.45 ^b,B,β^
After 48 h of marinating								
WBM + Mp + Lc	nd	5.85 ± 0.17 ^b,A,β^	nd	nd	nd	14.9 ± 0.47 ^c,A,β^	32.2 ± 0.96 ^c,A,β^	66.8 ± 0.95 ^d,B,φ^
WBM + Mp + Lc + ApBp	nd	4.34 ± 0.13 ^a,A,β^	nd	nd	nd	6.58 ± 0.19 ^a,A,β^	19.4 ± 0.58 ^a,A,β^	57.6 ± 0.99 ^c,A,β^
WBM + Mp + Lc + BcBp	nd	4.42 ± 0.13 ^a,A,β^	nd	nd	nd	nd	24.0 ± 0.71 ^b,A,β^	57.7 ± 0.82 ^c,B,β^
WBM + Mp + Lu	nd	nd	nd	nd	nd	nd	nd	52.2 ± 0.87 ^b,B,β^
WBM + Mp + Lu + ApBp	nd	nd	nd	nd	nd	9.64 ± 0.28 ^b,B,β^	nd	53.8 ± 0.67 ^b,B,β^
WBM + Mp + Lu + BcBp	nd	nd	nd	nd	nd	14.2 ± 0.42 ^c,A,β^	nd	46.2 ± 0.84 ^a,A,β^

WBM-wooden breast meat; Mp-milk permeate; Lc-*Lc. casei*; Lu-*Liq. uvarum*; ApBp-apple by-products; BcBp-blackcurrant by-products; TRY-tryptamine; PHE-phenylethylamine; PUTR-putrescine; CAD-cadaverine; HIS-Hystamine; TYR-tyramine; SPRMD-spermidine; SPRM-spermine; nd-not detected; ^a–f^ Mean values followed by ta different superscript letter in the column are significantly different (*p* ≤ 0.05) between treatment groups for the same time duration; ^A,B^ Mean values followed by a different superscript letter in the column are significantly different (*p* ≤ 0.05) between treatment groups for different marination duration; ^φ,β^ Mean values followed by a different superscript letter in the column are significantly different from the control group (*p* ≤ 0.05); data expressed as mean value (*n* = 3) ± standard error (SE).

**Table 5:** Saturated fatty acid profile (mean values ± standard errors) (% of total fatty acid content) of broilers’ wooden breast meat.

Fatty Acid (% of Total Fatty Acid Content)									
Samples	C12:0	C14:0	C15:0	C16:0	C17:0	C18:0	C20:0	C21:0	SFA
WBM	0.500 ± 0.041 ^φ^	0.599 ± 0.041 ^φ^	0.059 ± 0.024 ^φ^	23.89 ± 0.12 ^φ^	0.126 ± 0.024 ^φ^	8.02 ± 0.41 ^φ^	0.051 ± 0.031 ^φ^	0.088 ± 0.080 ^φ^	33.34 ± 0.87 ^φ^
After 24 h of marinating									
WBM + Mp + Lc	0.251 ± 0.030 ^a,A,β^	0.582 ± 0.030 ^a,A,φ^	0.228 ± 0.122 ^ab,A,φ^	20.15 ± 0.31 ^a,A,β^	0.296 ± 0.101 ^a,A,β^	9.64 ± 1.33 ^c,A,φ^	0.066 ± 0.021 ^a,A,φ^	0.144 ± 0.020 ^a,A,φ^	31.37 ± 0.28 ^b,A,β^
WBM + Mp + Lc + ApBp	0.095 ± 0.021 ^a,A,β^	0.828 ± 0.074 ^b,B,β^	0.149 ± 0.065 ^a,A,φ^	19.99 ± 0.05 ^a,A,β^	0.175 ± 0.056 ^ab,A,φ^	8.45 ± 0.04 ^ab,B,φ^	0.059 ± 0.018 ^a,A,φ^	0.125 ± 0.035 ^a,A,φ^	29.88 ± 0.50 ^a,A,β^
WBM + Mp + Lc + BcBp	0.120 ± 0.014 ^a,A,β^	0.529 ± 0.040 ^a,A,φ^	0.058 ± 0.041 ^a,A,φ^	21.88 ± 0.05 ^c,A,β^	0.143 ± 0.075 ^ab,A,φ^	6.48 ± 0.22 ^a,A,β^	0.046 ± 0.014 ^a,A,φ^	0.138 ± 0.018 ^a,A,φ^	29.39 ± 0.14 ^a,A,β^
WBM + Mp + Lu	0.905 ± 0.100 ^b,A,β^	1.19 ± 0.08 ^c,B,β^	0.049 ± 0.010 ^a,A,φ^	22.17 ± 0.08 ^c,A,β^	0.054 ± 0.016 ^a,A,β^	6.75 ± 0.14 ^a,A,β^	0.028 ± 0.01 ^a,A,φ^	0.138 ± 0.041 ^a,A,φ^	31.28 ± 0.24 ^b,A,β^
WBM + Mp + Lu + ApBp	1.11 ± 0.110 ^c,B,β^	1.29 ± 0.01 ^c,B,β^	0.039 ± 0.017 ^a,A,φ^	20.69 ± 0.06 ^b,A,β^	0.094 ± 0.055 ^a,A,φ^	6.98 ± 0.15 ^ab,A,β^	0.024 ± 0.014 ^a,A,φ^	0.087 ± 0.018 ^a,Aφ^	30.32 ± 0.11 ^ab,Aβ^
WBM + Mp + Lu + BcBp	1.02 ± 0.094 ^bc,A,β^	1.21 ± 0.13 ^c,A,β^	0.029 ± 0.011 ^a,A,φ^	20.85 ± 0.11 ^b,A,β^	0.091 ± 0.014 ^a,A,β^	6.92 ± 0.11 ^ab,A,β^	0.055 ± 0.011 ^a,A,φ^	0.116 ± 0.014 ^a,A,φ^	30.28 ± 0.54 ^ab,A,β^
After 48 h of marinating									
WBM + Mp + Lc	0.228 ± 0.020 ^b,A,β^	0.401 ± 0.111 ^a,A,φ^	0.200 ± 0.101 ^a,A,φ^	20.48 ± 0.26 ^a,A,β^	0.153 ± 0.021 ^a,A,φ^	9.47 ± 1.48 ^b,A,φ^	0.060 ± 0.008 ^b,A,φ^	0.221 ± 0.080 ^a,A,φ^	31.21 ± 0.79 ^bc,A,β^
WBM + Mp + Lc + ApBp	0.073 ± 0.044 ^a,A,β^	0.496 ± 0.121 ^ab,A,φ^	0.120 ± 0.015 ^ab,A,β^	20.89 ± 0.05 ^b,B,β^	0.128 ± 0.078 ^a,A,φ^	7.67 ± 0.08 ^a,A,φ^	0.031 ± 0.005 ^a,A,φ^	0.162 ± 0.041 ^a,A,φ^	29.57 ± 0.32 ^a,A,β^
WBM + Mp + Lc + BcBp	0.120 ± 0.081 ^ab,A,β^	0.595 ± 0.094 ^ab,A,φ^	0.031 ± 0.017 ^a,A,φ^	22.41 ± 0.04 ^d,B,β^	0.148 ± 0.078 ^a,A,φ^	6.43 ± 0.09 ^a,A,β^	0.031 ± 0.004 ^a,A,φ^	0.154 ± 0.040 ^a,A,φ^	29.92 ± 0.72 ^ab,A,β^
WBM + Mp + Lu	0.661 ± 0.030 ^c,A,β^	0.780 ± 0.150 ^bc,A,φ^	0.020 ± 0.014 ^a,A,φ^	22.41 ± 0.08 ^d,B,β^	0.050 ± 0.023 ^a,A,β^	7.16 ± 0.08 ^a,B,φ^	0.027 ± 0.005 ^a,A,φ^	0.120 ± 0.022 ^a,A,φ^	31.23 ± 0.10 ^c,A,β^
WBM + Mp + Lu + ApBp	0.732 ± 0.090 ^cd,A,β^	1.07 ± 0.07 ^cd,A,β^	0.040 ± 0.018 ^a,A,φ^	20.65 ± 0.09 ^a,A,β^	0.085 ± 0.021 ^a,A,φ^	6.69 ± 0.01 ^a,A,β^	0.025 ± 0.003 ^a,A,φ^	0.148 ± 0.074 ^a,A,φ^	29.44 ± 0.21 ^a,A,β^
WBM + Mp + Lu + BcBp	0.864 ± 0.024 ^d,A,β^	1.15 ± 0.13 ^d,A,β^	0.024 ± 0.014 ^a,A,φ^	21.36 ± 0.05 ^c,B,β^	0.082 ± 0.006 ^a,A,φ^	7.51 ± 0.04 ^a,B,φ^	0.056 ± 0.012 ^b,A,φ^	0.128 ± 0.025 ^a,A,φ^	31.18 ± 0.23 ^ab,A,β^

WBM-wooden breast meat; Mp-milk permeate; Lc-*Lc. casei*; Lu*-Liq. uvarum*; ApBp-apple by-products; BcBp-blackcurrant by-products; 12:0-dodecanoic acid; C14:0-tetradecanoic acid; C15:0-pentadecanoic acid; C16:0-hexadecanoic acid; C17:0-heptadecanoic acid; C18:0-octadecanoic acid; C20:0-eicosanoic acid; C21:0-heneicosanoic acid; SFA-saturated fatty acids. ^a–d^ Mean values followed by a different superscript letter in the column are significantly different (*p* ≤ 0.05) between treatment groups for the same time duration; ^A,B^ Mean values followed by a different superscript letter in the column are significantly different (*p* ≤ 0.05) between treatment groups for different marination duration; ^φ,β^ Mean values followed by a different superscript letter in the column are significantly different from the control group (*p* ≤ 0.05); data expressed as mean value (*n* = 3) ± standard error (SE).

**Table 6:** Monounsaturated fatty acid profile (mean values ± standard errors) (% of total fatty acid content) of broilers’ wooden breast meat.

Fatty Acid (% of Total Fatty Acid Content)						
Samples	C14:1	C16:1 ω7	C18:1 ω9	C18:1*trans* ω7	C20:1 ω7	MUFA
WBM	0.085 ± 0.010 ^φ^	4.21 ± 0.21 ^φ^	33.94 ± 0.04 ^φ^	1.87 ± 0.03 ^φ^	0.256 ± 0.028 ^φ^	40.36 ± 0.26 ^φ^
After 24 h of marinating						
WBM + Mp + Lc	0.001 ± 0.001 ^a,A,β^	2.51 ± 0.01 ^a,A,β^	34.25 ± 0.04 ^b,B,β^	2.14 ± 0.11 ^ab,B,β^	0.225 ± 0.017 ^a,A,φ^	39.12 ± 0.28 ^ab,B,β^
WBM + Mp + Lc + ApBp	0.024 ± 0.002 ^b,B,β^	2.53 ± 0.00 ^a,A,β^	34.65 ± 0.03 ^c,A,β^	1.84 ± 0.05 ^ab,B,φ^	0.238 ± 0.001 ^a,A,φ^	39.29 ± 0.39 ^ab,A,β^
WBM + Mp + Lc + BcBp	0.075 ± 0.015 ^c,B,φ^	4.51 ± 0.25 ^c,A,φ^	36.64 ± 0.05 ^d,B,β^	2.25 ± 0.06 ^b,B,φ^	0.305 ± 0.002 ^b,A,φ^	43.78 ± 0.11 ^d,B,β^
WBM + Mp + Lu	0.068 ± 0.006 ^c,B,φ^	4.20 ± 0.21 ^c,A,φ^	34.27 ± 0.06 ^b,A,β^	1.77 ± 0.04 ^ab,A,β^	0.255 ± 0.012 ^a,A,φ^	40.56 ± 0.27 ^c,A,φ^
WBM + Mp + Lu + ApBp	0.074 ± 0.002 ^c,B,φ^	3.03 ± 0.00 ^b,A,β^	33.93 ± 0.11 ^a,A,φ^	1.74 ± 0.05 ^ab,B,β^	0.246 ± 0.016 ^a,A,φ^	39.02 ± 0.05 ^a,A,β^
WBM + Mp + Lu + BcBp	0.040 ± 0.003 ^b,A,β^	3.15 ± 0.01 ^b,B,β^	34.73 ± 0.02 ^c,B,β^	1.55 ± 0.01 ^a,B,β^	0.213 ± 0.034 ^a,A,φ^	39.69 ± 0.04 ^b,B,β^
After 48 h of marinating						
WBM + Mp + Lc	0.001 ± 0.001 ^a,A,β^	2.64 ± 0.01 ^a,B,β^	33.85 ± 0.18 ^a,A,φ^	1.39 ± 0.02 ^a,A,β^	0.280 ± 0.032 ^bc,B,φ^	38.16 ± 0.19 ^a,A,β^
WBM + Mp + Lc + ApBp	0.007 ± 0.002 ^b,A,β^	2.97 ± 0.02 ^b,B,β^	34.68 ± 0.04 ^b,A,β^	1.66 ± 0.08 ^b,A,β^	0.232 ± 0.013 ^ab,A,φ^	39.55 ± 0.44 ^b,A,φ^
WBM + Mp + Lc + BcBp	0.006 ± 0.001 ^ab,A,β^	4.42 ± 0.21 ^d,A,φ^	36.10 ± 0.03 ^d,A,β^	1.90 ± 0.02 ^c,A,φ^	0.361 ± 0.009 ^d,B,β^	42.79 ± 0.16 ^d,A,β^
WBM + Mp + Lu	0.010 ± 0.003 ^b,A,β^	4.05 ± 0.02 ^c,A,φ^	34.97 ± 0.06 ^c,B,β^	1.94 ± 0.17 ^c,A,φ^	0.306 ± 0.005 ^c,B,φ^	41.27 ± 0.24 ^c,B,β^
WBM + Mp + Lu + ApBp	0.011 ± 0.003 ^b,A,β^	3.06 ± 0.01 ^b,A,β^	34.67 ± 0.02 ^b,B,β^	1.57 ± 0.04 ^b,A,β^	0.303 ± 0.004 ^c,B,β^	39.60 ± 0.22 ^b,B,β^
WBM + Mp + Lu + BcBp	0.008 ± 0.001 ^b,B,β^	2.83 ± 0.01 ^ab,A,β^	33.85 ± 0.11 ^a,A,φ^	1.40 ± 0.03 ^a,A,β^	0.214 ± 0.031 ^a,A,φ^	38.30 ± 0.24 ^a,A,β^

WBM-wooden breast meat; Mp-milk permeate; Lc-*Lc. casei*; Lu*-Liq. uvarum*; ApBp-apple by-products; BcBp-blackcurrant by-products; C14:1-tetradecenoic acid; C16:1 ω7-*cis*-9-hexadecenoic acid; C18:1 ω9-*cis*-9-octadecenoic acid; C18:*1 trans* ω7-*trans*-11-octadecenoic acid; C20:1 ω7-*cis*-13-eicosenoic acid; MUFA-monounsaturated fatty acids. ^a–d^ Mean values followed by a different superscript letter in the column are significantly different (*p* ≤ 0.05) between treatment groups for the same time duration; ^A,B^ Mean values followed by a different superscript letter in the column are significantly different (*p* ≤ 0.05) between treatment groups for different marination duration; ^φ,β^ Mean values followed by a different superscript letter in the column are significantly different from the control group (*p* ≤ 0.05); data expressed as mean value (*n* = 3) ± standard error (SE).

**Table 7:** Polyunsaturated fatty acid profile (mean values ± standard errors) (% of total fatty acid content) in broilers’ wooden breast meat.

Fatty Acid (% of Total Fatty Acid Content)								
Samples	C18:2 ω6	C18:3α ω3	C18:3γ ω6	C20:2 ω6	C20:3 ω6	C20:4 ω6	C20:5 ω3	PUFA
WBM	24.20 ± 0.05 ^a,A,φ^	1.059 ± 0.023 ^c,A,φ^	0.301 ± 0.030 ^b,A,φ^	0.095 ± 0.007 ^a,A,φ^	0.123 ± 0.012 ^a,A,φ^	0.461 ± 0.010 ^b,A,φ^	0.058 ± 0.002 ^a,A,φ^	26.30 ± 0.07 ^a,A,φ^
After 24 h of marinating								
WBM + Mp + Lc	27.29 ± 0.03 ^c,A,β^	0.941 ± 0.040 ^b,B,β^	0.159 ± 0.014 ^a,A,β^	0.273 ± 0.014 ^b,A,β^	0.201 ± 0.035 ^b,A,β^	0.555 ± 0.016 ^c,A,β^	0.098 ± 0.001 ^a,B,β^	29.52 ± 0.15 ^c,A,β^
WBM + Mp + Lc + ApBp	28.78 ± 0.05 ^f,A,β^	0.893 ± 0.010 ^ab,A,β^	0.198 ± 0.034 ^ab,A,β^	0.226 ± 0.014 ^a,B,β^	0.172 ± 0.009 ^b,A,β^	0.469 ± 0.001 ^b,A,φ^	0.094 ± 0.017 ^a,B,φ^	30.83 ± 0.13 ^e,A,β^
WBM + Mp + Lc + BcBp	25.06 ± 0.041 ^a,A,β^	0.822 ± 0.031 ^a,A,β^	0.191 ± 0.039 ^ab,B,β^	0.203 ± 0.004 ^a,B,β^	0.189 ± 0.003 ^b,A,β^	0.266 ± 0.001 ^a,A,β^	0.097 ± 0.009 ^a,B,β^	26.83 ± 0.09 ^a,A,β^
WBM + Mp + Lu	25.94 ± 0.03 ^b,B,β^	0.819 ± 0.020 ^a,A,β^	0.317 ± 0.021 ^c,A,φ^	0.215 ± 0.022 ^a,B,β^	0.190 ± 0.016 ^b,A,β^	0.612 ± 0.021 ^d,B,β^	0.063 ± 0.016 ^a,A,φ^	28.16 ± 0.10 ^b,B,β^
WBM + Mp + Lu + ApBp	28.51 ± 0.07 ^e,A,β^	1.03 ± 0.04 ^c,A,φ^	0.260 ± 0.021 ^bc,B,φ^	0.189 ± 0.017 ^a,A,β^	0.121 ± 0.014 ^a,A,φ^	0.468 ± 0.029 ^b,A,φ^	0.072 ± 0.017 ^a,A,φ^	30.65 ± 0.09 ^e,A,β^
WBM + Mp + Lu + BcBp	27.91 ± 0.085 ^d,A,β^	0.838 ± 0.020 ^a,A,β^	0.175 ± 0.014 ^a,A,β^	0.312 ± 0.011 ^b,B,β^	0.161 ± 0.009 ^ab,A,β^	0.555 ± 0.010 ^c,A,β^	0.075 ± 0.012 ^a,A,φ^	30.03 ± 0.19 ^d,A,β^
After 48 h of marinating								
WBM + Mp + Lc	28.10 ± 0.06 ^b,B,β^	0.804 ± 0.011 ^a,A,β^	0.224 ± 0.028 ^c,B,β^	0.359 ± 0.021 ^d,B,β^	0.307 ± 0.021 ^b,B,β^	0.779 ± 0.014 ^d,B,β^	0.054 ± 0.002 ^a,A,φ^	30.63 ± 0.13 ^bc,B,β^
WBM + Mp + Lc + ApBp	28.72 ± 0.03 ^d,A,β^	0.888 ± 0.013 ^b,A,β^	0.181 ± 0.015 ^bc,B,β^	0.143 ± 0.010 ^a,A,β^	0.224 ± 0.014 ^a,B,β^	0.658 ± 0.014 ^c,B,β^	0.062 ± 0.016 ^a,A,φ^	30.88 ± 0.21 ^cd,A,β^
WBM + Mp + Lc + BcBp	25.37 ± 0.03 ^a,B,β^	0.802 ± 0.010 ^a,A,β^	0.111 ± 0.008 ^a,A,β^	0.171 ± 0.002 ^a,A,β^	0.213 ± 0.011 ^a,B,β^	0.576 ± 0.007 ^b,B,β^	0.052 ± 0.014 ^a,A,φ^	27.29 ± 0.04 ^a,B,β^
WBM + Mp + Lu	25.48 ± 0.06 ^a,A,β^	0.810 ± 0.041 ^a,A,β^	0.290 ± 0.015 ^d,A,φ^	0.170 ± 0.013 ^a,A,β^	0.200 ± 0.032 ^a,A,β^	0.512 ± 0.041 ^a,A,φ^	0.040 ± 0.015 ^a,A,φ^	27.50 ± 0.10 ^a,A,β^
WBM + Mp + Lu + ApBp	28.72 ± 0.02 ^d,B,β^	1.08 ± 0.07 ^c,A,φ^	0.158 ± 0.022 ^ab,A,β^	0.232 ± 0.015 ^b,B,β^	0.207 ± 0.010 ^a,B,β^	0.510 ± 0.018 ^a,A,φ^	0.041 ± 0.013 ^a,A,φ^	30.95 ± 0.07 ^d,A,β^
WBM + Mp + Lu + BcBp	28.43 ± 0.03 ^c,B,β^	0.818 ± 0.031 ^a,A,β^	0.185 ± 0.010 ^bc,A,β^	0.278 ± 0.008 ^c,A,β^	0.180 ± 0.017 ^a,A,β^	0.580 ± 0.013 ^b,A,β^	0.050 ± 0.010 ^a,A,φ^	30.54 ± 0.06 ^b,B,β^

WBM-wooden breast meat; Mp-milk permeate; Lc-*Lc. casei*; Lu*-Liq. uvarum*; ApBp-apple by-products; BcBp-blackcurrant by-products; C18:2 ω6-*cis*-9,12-octadecadienoic acid, C18:3α ω3-*cis*-9,12,15-octadecatrienoic acid, C18:3γ ω6-*cis*-6,9,12- octadecatrienoic acid, C20:2 ω6-*cis*-11,4-eicosadienoic acid, C20:3 ω6-*cis*-11,14,17-eicosatrienoic acid, C20:4 ω6-*cis*-5,8,11,14-eicosatetraenoic acid, C20:5 ω3-*cis*-5,8,11,14,17-eicosapentaenoic acid; PUFA-polyunsaturated fatty acids. ^a–f^ Mean values followed by a different superscript letter in the column are significantly different (*p* ≤ 0.05) between treatment groups for the same time duration; ^A,B^ Mean values followed by a different superscript letter in the column are significantly different (*p* ≤ 0.05) between treatment groups for different marination duration; ^φ,β^ Mean values followed by a different superscript letter in the column are significantly different from the control group (*p* ≤ 0.05); data expressed as the mean value (*n* = 3) ± standard error (SE).

## Data Availability

The original contributions presented in the study are included in the article, further inquiries can be directed to the corresponding authors.

## Abbreviations

Analysis of variance (ANOVA); Apple processing by-product (ApBp); Ash content (AC); Biogenic amine (BA); Blackcurrant processing by-product (BcBp); Cadaverine (CAD); cis-11;14;17-Eicosatrienoic acid (C20:3 ω6); cis-11;4-Eicosadienoic acid (C20:2 ω6); cis-13-Eicosenoic acid (C20:1 ω7); cis-5;8;11;14;17-Eicosapentaenoic acid (C20:5 ω3); cis-5;8;11;14-Eicosatetraenoic acid (C20:4 ω6); cis-6;9;12- Octadecatrienoic acid (C18:3γ ω6); cis-9;12;15-Octadecatrienoic acid (C18:3α ω3); cis-9;12-Octadecadienoic acid (C18:2 ω6); cis-9-Hexadecenoic acid (C16:1 ω7); cis-9-Octadecenoic acid (C18:1 ω9); Colony-forming units (CFU); Cooking loss (CL); Dodecanoic acid (C12:0); Drip loss (DL); Dry-matter (DM); Eicosanoic acid (C20:0); Fat content (FC); Fatty acid composition (FA); Fatty acid methyl esters (FAME); Heneicosanoic acid (C21:0); Heptadecanoic acid (C17:0); Hexadecanoic acid (C16:0); Highly unsaturated fatty acids (HUFA); Histamine (HIS); Lactic acid bacteria (LAB); Lacticaseibacillus casei (Lc); Liquorilactobacillus uvarum (Lu); Milk permeate (MP); Mold/yeast viable counts (M/Y); Monounsaturated fatty acids (MUFA); Not detected (nd); Octadecanoic acid (C18:0); Pentadecanoic acid (C15:0); Phenylethylamine (PHE); Polyunsaturated fatty acids (PUFA); Protein content (PC); Putrescine (PUTR); Saturated fatty acids (SFA); Shear-force (SF); Spermidine (SPRMD); Spermine (SPRM); Standard error (SE); Tetra-decanoic acid (C14:0); Tetra-decenoic acid (C14:1); Total bacterial viable counts (TBC); Total enterobacteria viable counts (TEC); Total titratable acidity (TTA); trans-11-Octadecenoic acid (C18:1trans ω7); Tryptamine (TRY); Tukey’s-honest significant difference (Tukey-HSD); Tyramine (TYR); Water-holding capacity (WHC); Wooden breast (WB); Wooden breast meat (WBM).
